# Control of androgen receptor action by a novel nuclear receptor binding motif in Bag-1L

**DOI:** 10.1186/1756-8935-6-S1-P13

**Published:** 2013-03-18

**Authors:** Laura Cato, Katja Jehle, Antje Neeb, Andrew CB Cato, Myles Brown

**Affiliations:** 1 Department of Medical Oncology, Dana-Farber Cancer Institute and Harvard Medical School, Boston, USA; 2 Center for Functional Cancer Epigenetics, Dana-Farber Cancer Institute, Boston, USA; 3 Institute of Toxicology and Genetics, Karlsruhe Institute of Technology KIT, Eggenstein-Leopoldshafen, Germany

## Background

The androgen receptor (AR) is an important determinant of normal and malignant prostate growth. Therefore, a good understanding of the factors that regulate the transactivation of the AR is essential and could provide a better strategy to control prostate tumor growth, particularly in patients suffering from the hormone-refractory and the advanced stages of the disease. However, transcriptional activation by the AR is a complex and orchestrated process requiring multiple coregulators. Several coregulators have already been identified, including proteasome components, chromatin-re-modeling complexes and heat shock proteins. Others however remain uncharacterized and their role in AR transactivation is poorly understood. One such coregulator is the nuclear-resident, AR co-activator, Bag-1L. Overexpression of Bag-1L and the amplification of its gene have been reported in the hormone-refractory and metastatic stages of prostate cancer and in androgen-independent prostate cancer cells (AIPC). However the exact mechanism of Bag-1L-mediated regulation of AR action in prostate cancer is unclear.

## Materials and methods

To confirm the contribution of Bag-1L to AR response in prostate cancer, we have downregulated the expression of Bag-1L in the androgen-dependent prostate cancer cell line LNCaP using RNAi. Subsequently we performed genome-wide mapping of Bag-1L, using genome-wide chromatin immunoprecipitation (ChIP-seq). Lastly, the ability of AR and Bag-1L to interact with one another was assessed by domain mapping experiments using GST pulldown assays.

## Results

Here we show that Bag-1L depletion by RNA interference in the prostate cell line LNCaP significantly reduces the hormone response of several AR-target genes. In agreement, Bag-1L and AR co-localize to a large number of regulatory regions of AR-target genes as identified by ChIP-sequencing. Furthermore, domain mapping experiments identified the first 128 N-terminal amino acids of Bag-1L as necessary for enhancing the transactivation function of the receptor. This sequence contains a duplication of a GARRPR motif, which we identified as the main interaction site between Bag-1L and AR. We were able to further confirm the importance of this motif by amino acid substitutions of the sequence, which impairs the AR and Bag-1L-mediated AR transactivation. Intriguingly, overexpression of the N-terminal sequence of Bag-1L encompassing the conserved hexapeptide sequences exerts a dominant negative effect on androgen-mediated gene expression and androgen-dependent tumor growth. The GARRPR was also identified in other regulators of AR activity, such as Huntington associated protein 1, nuclear receptor coactivator 4 and p21-activated kinase 6.

## Conclusions

We have identified a novel AR binding motif different from the previously described LXXLL and FXXLF sequences of other coactivators of AR. In the long-term we propose to use this knowledge to design novel drugs targeting this interaction site for the therapeutic intervention of prostate cancer.

